# Novel role of the clustered miR‐23b‐3p and miR‐27b‐3p in enhanced expression of fibrosis‐associated genes by targeting TGFBR3 in atrial fibroblasts

**DOI:** 10.1111/jcmm.14211

**Published:** 2019-02-07

**Authors:** Zhenzhen Yang, Zhen Xiao, Huiming Guo, Xianhong Fang, Jingnan Liang, Jiening Zhu, Jing Yang, Hui Li, Rong Pan, Shujing Yuan, Wenyan Dong, Xi‐Long Zheng, Shulin Wu, Zhixin Shan

**Affiliations:** ^1^ Guangdong Provincial Key Laboratory of Clinical Pharmacology Guangdong Cardiovascular Institute Guangzhou China; ^2^ Research Center of Medical Sciences Guangdong Provincial People's Hospital, Guangdong Academy of Medical Sciences Guangzhou China; ^3^ School of Medicine South China University of Technology Guangzhou China; ^4^ School of Pharmacy Southern Medical University Guangzhou China; ^5^ Guangzhou Women and Children's Medical Center, Institute of Pediatrics, Guangzhou Medical University Guangzhou China; ^6^ Department of Biochemistry & Molecular Biology The Libin Cardiovascular Institute of Alberta, The University of Calgary Calgary Alberta Canada

**Keywords:** atrial fibrosis, human atrial fibroblast, microRNA‐23b‐3p, microRNA‐27b‐3p, Smad3, TGFBR3

## Abstract

Atrial fibrillation (AF) is the most common type of arrhythmia in cardiovascular diseases. Atrial fibrosis is an important pathophysiological contributor to AF. This study aimed to investigate the role of the clustered miR‐23b‐3p and miR‐27b‐3p in atrial fibrosis. Human atrial fibroblasts (HAFs) were isolated from atrial appendage tissue of patients with sinus rhythm. A cell model of atrial fibrosis was achieved in Ang‐II‐induced HAFs. Cell proliferation and migration were detected. We found that miR‐23b‐3p and miR‐27b‐3p were markedly increased in atrial appendage tissues of AF patients and in Ang‐II‐treated HAFs. Overexpression of miR‐23b‐3p and miR‐27b‐3p enhanced the expression of collagen, type I, alpha 1 (COL1A1), COL3A1 and ACTA2 in HAFs without significant effects on their proliferation and migration. Luciferase assay showed that miR‐23b‐3p and miR‐27b‐3p targeted two different sites in 3ʹ‐UTR of transforming growth factor (TGF)‐β1 receptor 3 (TGFBR3) respectively. Consistently, TGFBR3 siRNA could increase fibrosis‐related genes expression, along with the Smad1 inactivation and Smad3 activation in HAFs. Additionally, overexpression of TGFBR3 could alleviate the increase of COL1A1, COL3A1 and ACTA2 in HAFs after transfection with miR‐23b‐3p and miR‐27b‐3p respectively. Moreover, Smad3 was activated in HAFs in response to Ang‐II treatment and inactivation of Smad3 attenuated up‐regulation of miR‐23b‐3p and miR‐27b‐3p in Ang‐II‐treated HAFs. Taken together, these results suggest that the clustered miR‐23b‐3p and miR‐27b‐3p consistently promote atrial fibrosis by targeting TGFBR3 to activate Smad3 signalling in HAFs, suggesting that miR‐23b‐3p and miR‐27b‐3p are potential therapeutic targets for atrial fibrosis.

## 1 INTRODUCTION

Atrial fibrillation (AF) is the most common type of arrhythmia in cardiovascular diseases. The incidence and prevalence of AF increase as the population ages.^1^ Atrial electrical remodeling and structural remodeling are important aspects of the pathogenesis of AF.^2^ Although much progress has been made during the past several decades, the mechanisms and pathophysiology of AF are still not well elucidated. Atrial fibrosis has been known as an important pathophysiological contributor and has been linked to AF recurrences and resistance to clinical therapy. Increasing evidence indicates that fibrosis plays a central role in stabilizing the re‐entrant drivers that maintain the arrhythmia.^3^, ^4^, ^5^ To prevent AF through suppressing fibrosis has been supported experimentally and accepted as a promising potential therapeutic strategy for AF patients.^6^

Non‐coding RNAs (ncRNAs) comprise over 95% of the total RNA transcripts in eukaryotic cells.^7^ Recently, more than 5000 miRNAs have been discovered in the human genome.^8^ MicroRNA (miRNA) is an endogenous non‐coding RNA with about 22 nucleotides and is involved in a variety of biological processes and diseases by inhibiting the protein translation process of the target gene.^9^, ^10^, ^11^ It has been found that miRNA can also participate in the development of atrial fibrillation through fibrosis‐related target genes. For example, miR‐21 is markedly up‐regulated in AF models and involved in multiple pro‐fibrotic pathways by targeting Spry1,^12^ PIAS3,^13^ Smad7^14^ and Jagged1^15^ respectively. However, miR‐26 down‐regulation in AF enhances atrial fibrosis by targeting TRPC3 to increase Ca^2+^ entry and activate proliferation and differentiation of fibroblasts^16^ and also by targeting KCNJ2 to enhance *I*_K1 _and atrial fibroblast proliferation.^17^

MiR‐23b‐3p and miR‐27b‐3p are located within a single transcription unit from a microRNA cluster on human chromosome 9 (www.mirdb.org). Inhibition of miR‐23b could alleviate cardiac fibrosis in caecal ligation puncture (CLP)‐induced late sepsis by targeting TGIF1 and PTEN to activate TGF‐β1/Smad2/3 signalling and AKT/N‐Cadherin signalling in cardiac fibroblasts respectively.^18^ The effects of miR‐27b on cardiac fibrosis are controversial. Overexpression of miR‐27b in cardiomyocytes was reported to contribute to cardiac dysfunction with enhanced myocardial fibrosis by targeting PPAR‐γ^19^ and MMP13^20^ respectively. However, a recent study showed that atrial overexpression of miR‐27b attenuated angiotensin II‐induced atrial fibrosis by targeting ALK5.^21^ To date, the role and mechanism of miR‐23b‐3p and miR‐27b‐3p in atrial fibrosis are not well understood.

In light of its essential role in the pathogenesis of fibrosis, TGF‐β has emerged as an attractive therapeutic target.^22^ TGF‐β1 receptor 3(TGFBR3) is negatively regulated by TGF‐β1 in multiple cell types and mediates TGF‐β superfamily ligand‐dependent as well as ligand‐independent signalling to both Smad and non‐Smad signalling pathways.^23^ A recent study showed that TGFBR3 inhibited TGF‐β1 expression, Smad2/3 activation and TGFBR1‐TGFBR2 complex formation and collagen production in cardiac fibroblasts.^24^ However, it still not known whether TGFBR3 can be modulated by miR‐23b‐3p and miR‐27b‐3p in atrial fibroblasts.

In this study, we investigated the expression of miR‐23b‐3p and miR‐27b‐3p in atrial appendages of patients with rheumatic AF and in HAFs treated with Ang‐II. Based on bio‐informatic analysis, we speculated that TGFBR3 is a target of miR‐23b‐3p and miR‐27b‐3p and further validated their interactions in this study. By illustrating the underlying mechanism that miR‐23b‐3p and miR‐27b‐3p facilitate atrial fibrosis progression, this study will provide new insight into atrial fibrosis target therapy.

## 2 MATERIALS AND METHODS

### 2.1 Ethics statement

The surgically removed right atrial appendages were obtained from nine patients with rheumatic atrial fibrillation (AF) (aged 40‐65 years, a maximal treadmill exercise test showing no ischaemia and without evidence of hypertension, coronary artery disease, congestive heart failure, diabetic complications) and seven age‐sex matched patients with sinus rhythm (SR). The clinical investigation was conducted according to the principles expressed in the Declaration of Helsinki and approved by the Research Ethics Committee of Guangdong General Hospital (Guangzhou, China). All patients gave written consents to participate in this study and were told to follow only conventional medical treatments without additional medication.

### 2.2 Histological analysis

Formalin‐fixed human atrial appendage specimens were embedded in paraffin and cut into 4 μm‐thick sections. Tissue sections were mounted on the regular glass slides and stained with Masson's trichrome for histological examination. For the collagen volume fraction (CVF) analysis in the atrial appendages, five separate views (original magnification ×400) were selected for assessment of CVF with the following formula: CVF = collagen area/total area.

### 2.3 Isolation, culture and treatment of human atrial fibroblasts (HAFs)

Human atrial fibroblasts (HAFs) were isolated from atrial appendage tissue of patients with SR. The atrial appendage tissue was digested using 0.25% trypsin plus 20 IU/mL DNase. HAFs were separated from cardiomyocytes using gravity separation and then grown to confluency in 10‐cm cell culture dishes in the growth media (DMEM/LG 10% FBS, 1% penicillin and 1% streptomycin) at 37°C in humid air with 5% CO_2_. HAFs from the fourth to sixth passage were used for the experiments. The miR‐23b‐3p mimic, miR‐27b‐3p mimic and TGFBR3 siRNA (50 nmol/L each, RiboBio, Guangzhou, China) were transfected into HAFs using oligofectamine reagent (Invitrogen, Carlsbad, CA, USA). HAFs were then incubated with DMEM/LG 1% FBS overnight before treatment with 10 μmol/L Ang‐II (Sigma‐Aldrich, St. Louis, MO, USA). As indicated, HAFs were infected with the following recombinant adenovirus respectively: rAd‐GFP, rAd‐TGFBR3 adenovirus (MOI 5).

### 2.4 Fluorescence immunohistochemistry (FIHC) assay

Cultured HAFs were washed in phosphate‐buffered saline (PBS), fixed for 10 minutes in 3.7% formaldehyde and permeabilized with 0.1% Triton X‐100 for 10 minutes. Monolayers were then washed in blocking solution and incubated with α‐SMA antibody (Abcam) overnight at 4°C. The monolayers were then washed again and incubated with Alexa Fluor^®^488dye‐conjugated IgG (Molecular Probes, Eugene, OR, USA) for 1 hour at room temperature. The nuclei were stained by incubating cells with 40, 6‐diamidino‐2‐phenylindole (DAPI). Confocal micrographs were obtained using a Leica SP5 confocal microscope (Leica, Mannheim, Germany). The fluorescence intensity analysis was performed with the LAS AF‐TCS SP5 imaging software.

### 2.5 Cell proliferation and migration assay

According to the manufacturer's instructions, CCK8 and Edu staining assays were conducted to determine the proliferation capacity of HAFs. Trans‐well migration assay was performed to assess the migration of HAFs. Briefly, HAFs were cultured in the upper chamber with a higher concentration of foetal bovine serum in the media in the lower chamber. The number of HAFs migrating into the lower chamber was counted to evaluate the migration of HAFs.

### 2.6 Quantitative mRNA and miRNA measurements

For detection of mRNA expression of coding genes, the first‐strand cDNA was generated from 1.5 μg total RNA using a mixture of oligo (dT)_15_ and random primers with superscript reverse transcriptase (Invitrogen, Carlsbad, CA). Quantitative reverse‐transcription PCR (RT‐qPCR) for miR‐23b‐3p and ‐27b‐3p was performed on cDNA generated from 0.5 μg of total RNA according to the manufacturer's protocol (Ribobio, China). To normalize RNA content, U6 was used for miRNA template normalization and GAPDH was used for coding gene template normalization. PCR was performed with the ViiA7 Quantitative PCR System (Applied Biosystems, Carlsbad, CA). The 2^–ΔΔCt^ method was used to calculate the relative expression levels of the concerned coding genes and miRNAs.

### 2.7 Western blot assay

Protein extracts (40 μg each) were separated using 12% (wt./vol.) SDS‐PAGE, transferred onto a polyvinylidene fluoride (PVDF) membrane and probed with respective antibodies for ACAT2 (Abcam), COL1A1 and COL3A1 (Proteintech, Chicago, IL, USA), p‐Smad3, Smad3 (Cell Signaling Technology, Beverly, MA, USA) overnight at 4°C. The membranes were then washed extensively with TBS/T and incubated with a horseradish peroxidase (HRP)‐conjugated secondary antibody (Santa Cruz) for 1 hour at room temperature. The protein was visualized using the ECL Plus detection system (GE Healthcare, WI, USA). As internal controls, the membranes were also immunoblotted with anti‐GAPDH and anti‐Lamin B1 antibody respectively (Santa Cruz).

### 2.8 Dual luciferase assay

The recombinant luciferase reporter plasmids containing sequences of potential miR‐23b‐3p and miR‐27b‐3p binding sites in 3ʹ UTR of TGFBR3 gene were constructed. Using a site‐directed mutagenesis kit (TransGen, Beijing, China), miR‐23b‐3p and miR‐27b‐3p complementary binding sequence AGUGU was replaced with ACACA to construct recombinant luciferase reporter plasmids containing the mutant binding sequences of miR‐23b‐3p and miR‐27b‐3p respectively.

Human embryonic kidney (HEK) 293 cells (3×10^5^ cells/well in 12‐well plate) were cotransfected with 200 ng of recombinant luciferase reporter plasmid, 50 nmol/L miR‐23b‐3p and miR‐27b‐3p and 10 ng of pRL‐TK as an internal control (Promega, Madison, WI). Activities of firefly luciferase (FL) and Renilla luciferase (RL) were measured 24 hours after transfection and the relative ratio of the FL/RL was used to indicate the knockdown of TGFBR3 by miR‐23b‐3p and miR‐27b‐3p respectively.

### 2.9 Statistical analysis

Data are presented as the mean ± the standard error of the mean (SEM). In each experiment, all determinations were performed at least in triplicate. Statistical significance between the two measurements was determined using the two‐tailed unpaired Student's *t* test and among groups, it was determined using one‐way ANOVA. A value of *P* < 0.05 was considered to be significant.

## 3 RESULTS

### 3.1 miR‐23b‐3p and miR‐27b‐3p are up‐regulated in atrial appendage tissue of AF patients

**Figure 1:**
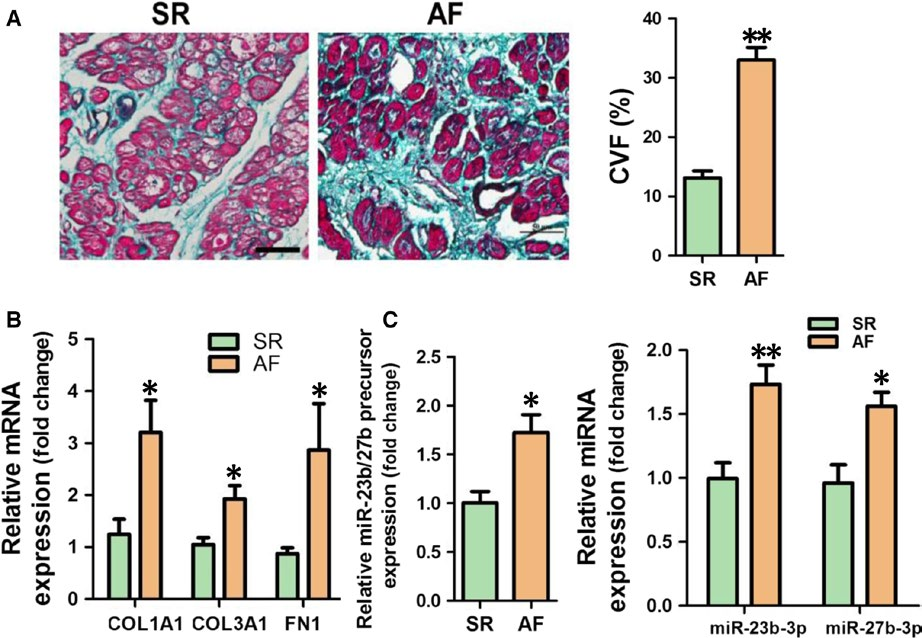
Up‐regulation of miR‐23b‐3p and miR‐27b‐3p in atrial appendage tissues of AF patients. A, Masson trichrome staining assay (scale bar is 50 μm). B, Col1a1, Col3a1 and FN1 mRNA expression were detected by RT‐qPCR assay in the atrial appendages of AF patients. C, Expression of miR‐23b/27b precursor, miR‐23b‐3p and miR‐27b‐3p was determined using RT‐qPCR assay in the atrial appendages of AF patients. Data are shown as mean ± SEM (n = 7‐9). *P < 0.05, **P < 0.01 vs SR control

Results of Masson trichrome staining revealed that internal fibrosis was markedly increased in the atrial appendages of AF patients (Figure 1A). We detected mRNA expression of Col1a1, Col3a1 and FN1 in atrial appendages of patients with SR or AF. The RT‐qPCR results showed that mRNA expression of the above fibrosis‐related genes was significantly up‐regulated in atrial appendages of AF patients (*P* < 0.05 respectively) (Figure 1B). Moreover, the inflammation‐related genes including IL‐1β and CRP, but not TNF‐α were significantly up‐regulated in atrial appendages of AF patients (Figure S1). However, no significant changes of RAAS‐associated genes, such as ACE1, ACE2 and Apelin were observed in the atrial appendages of AF patients (Figure S2). The expression levels of miR‐23b/27b precursor, miR‐23b‐3p and miR‐27b‐3p were up‐regulated in the atrial appendages of AF patients (Figure 1C), consistent with miRNA profiling data of atrial appendages from SR and AF patients (data not shown).

### 3.2 miR‐23b‐3p and miR‐27b‐3p are up‐regulated in Ang‐II‐treated human atrial fibroblasts (HAFs)

**Figure 2:**
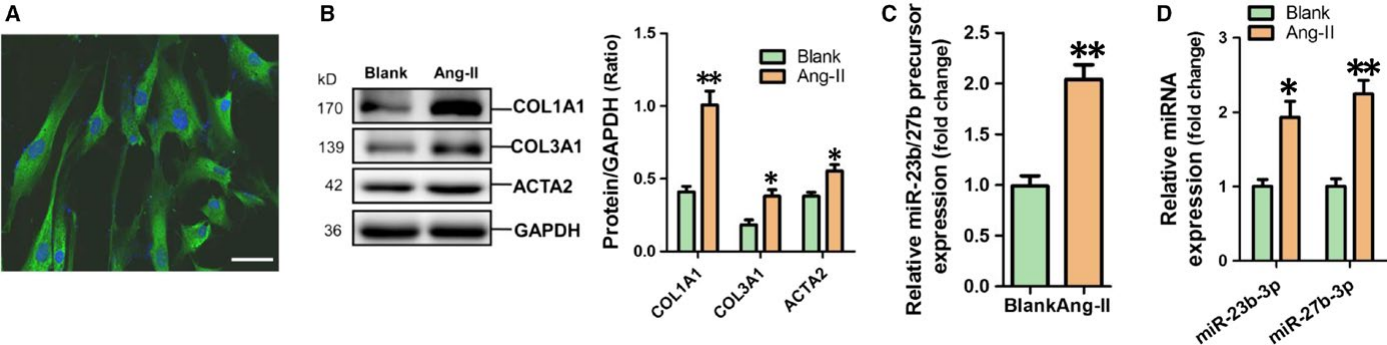
Up‐regulation of miR‐23b‐3p and miR‐27b‐3p in Ang‐II‐induced HAFs. A, Expression of ACTA2 in HAFs by immunofluorescent staining (scale bar is 50 μm). B, Col1a1, Col3a1 and ACTA2 expression was detected using western blot assay in Ang‐II‐induced HAFs. Expression of miR‐23b/27b precursor (C), miR‐23b‐3p and miR‐27b‐3p (D) was determined using RT‐qPCR assay in Ang‐II‐induced HAFs. Data are shown as mean ± SEM (n = 3). *P < 0.05, **P < 0.01 vs Blank control. Ang‐II was not used to treat HAFs in the Blank group

The immunofluorescent staining confirmed that HAFs were successfully isolated and cultured, with positive ACTA2 expression (Figure 2A). Fibrosis‐associated genes including COL1A1, COL3A1 and ACTA2, were significantly increased in Ang‐II‐treated HAFs (Figure 2B). Meanwhile, RT‐qPCR results showed that miR‐23b/27b precursor, miR‐23b‐3p and miR‐27b‐3p were markedly up‐regulated in HAFs exposed to Ang‐II (*P* < 0.05 respectively) (Figure 2C,D).

### 3.3 Effects of miR‐23b‐3p and miR‐27b‐3p on proliferation and migration of HAFs

**Figure 3:**
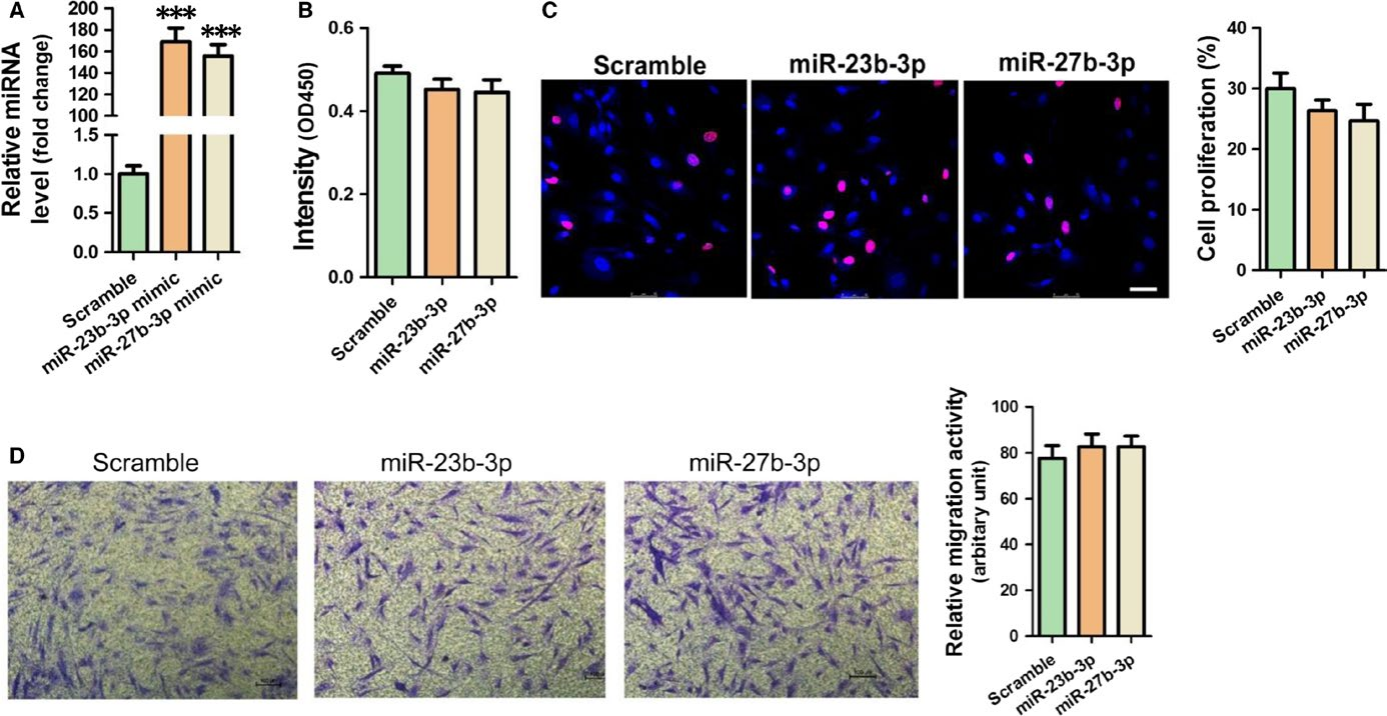
MiR‐23b‐3p and miR‐27b‐3p have no effects on proliferation and migration of HAFs. A, Determination of miR‐23b‐3p and miR‐27b‐3p in HAFs. Proliferation of HAFs was determined using CCK8 assay (B) and Edu assay (C) respectively (scale bar is 50 μm). D, Migration of HAFs was determined using trans‐well migration assay immunofluorescent staining (scale bar is 100 μm). Data are shown as mean ± SEM (n = 3). ***P < 0.001 vs Scramble control

MiR‐23b‐3p and miR‐27b‐3p mimic were transfected in HAFs using lipofectamine 2000 reagent. As expected, RT‐qPCR results indicated that miR‐23b‐3p and miR‐27b‐3p were efficiently delivered into HAFs (Figure 3A). CCK8 and Edu staining were performed to detect the proliferation of HAFs. Our results showed no significant differences in cell proliferation observed in HAFs transfected with miR‐23b‐3p and miR‐27b‐3p respectively (Figure 3B,C). Moreover, the trans‐well migration assay revealed no significant differences in cell migration among HAFs transfected with scramble, miR‐23b‐3p and miR‐27b‐3p respectively (Figure 3D).

### 3.4 MiR‐23b‐3p and miR‐27b‐3p enhance the expression of fibrosis‐related genes in HAFs

**Figure 4:**
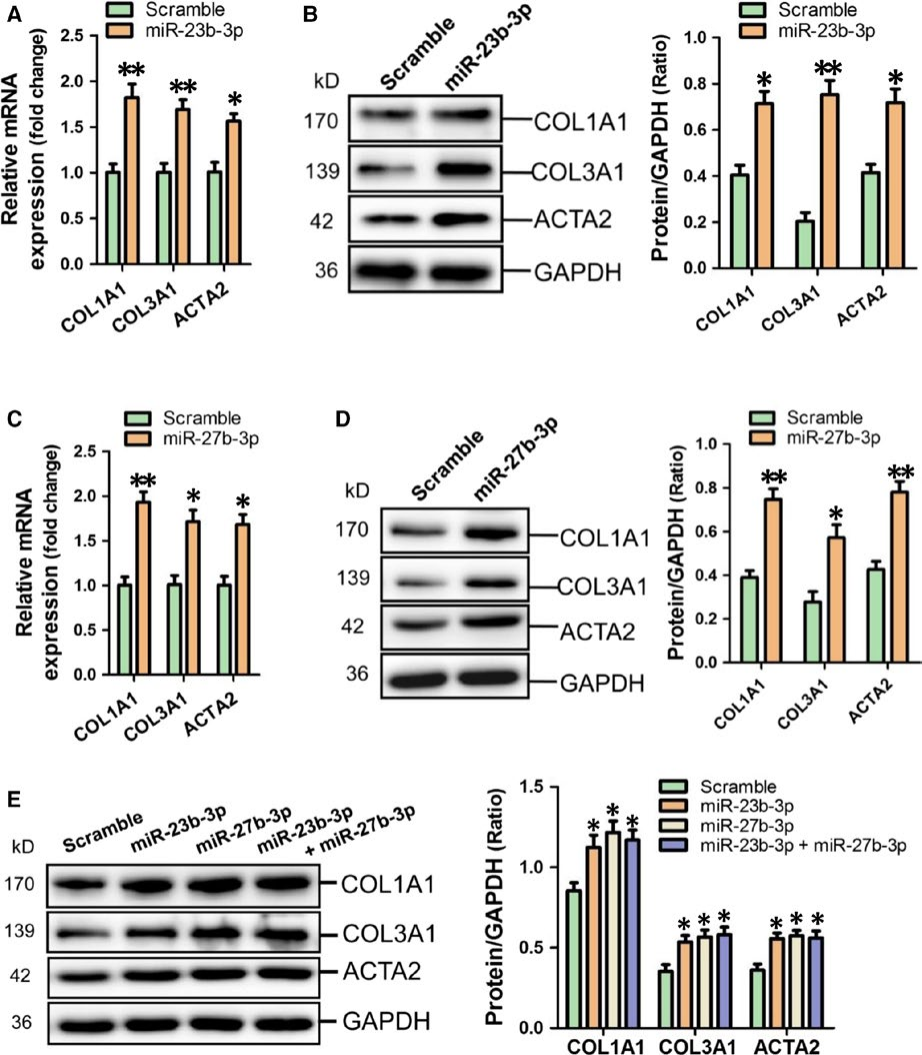
MiR‐23b‐3p and miR‐27b‐3p enhance the expression of fibrosis‐related gene in HAFs. COL1A1, COL3A1 and ACTA2 expression in miR‐23b‐3p‐modified HAFs was determined using RT‐qPCR (A) and western blot assay (B) respectively. The mRNA (C) and protein (D) expression of COL1A1, COL3A1 and ACTA2 in miR‐27b‐3p‐modified HAFs were determined using RT‐qPCR and western blot assay respectively. No synergetic increase of COL1A1, COL3A1 and ACTA2 expression was observed in HAFs with transfection of miR‐23b‐3p combined with miR‐27b‐3p (E). Data are shown as mean ± SEM (n = 3). *P < 0.05, **P < 0.01 vs Scramble control

To demonstrate the potential roles of miR‐23b‐3p and miR‐27b‐3p in atrial fibrosis, we determined fibrosis‐related gene expression in HAFs with overexpression of miR‐23b‐3p and miR‐27b‐3p respectively. Results of RT‐qPCR and western blot assay showed that miR‐23b‐3p efficiently enhanced COL1A1, COL3A1 and ACTA2 expression in HAFs (Figure 4A,B). Consistently, miR‐27b‐3p also increased the above fibrosis‐related gene expression at mRNA and protein level in HAFs (Figure 4C,D). However, no significant difference in COL1A1, COL3A1 and ACTA2 expression was observed in HAFs with transfection of miR‐23b‐3p together with miR‐27b‐3p compared with HAFs with overexpression of miR‐23b‐3p and miR‐27b‐3p respectively (Figure 4E).

### 3.5 MiR‐23b‐3p and miR‐27b‐3p interact with 3ʹ‐UTR of TGF‐β receptor type 3 (TGFBR3)

**Figure 5:**
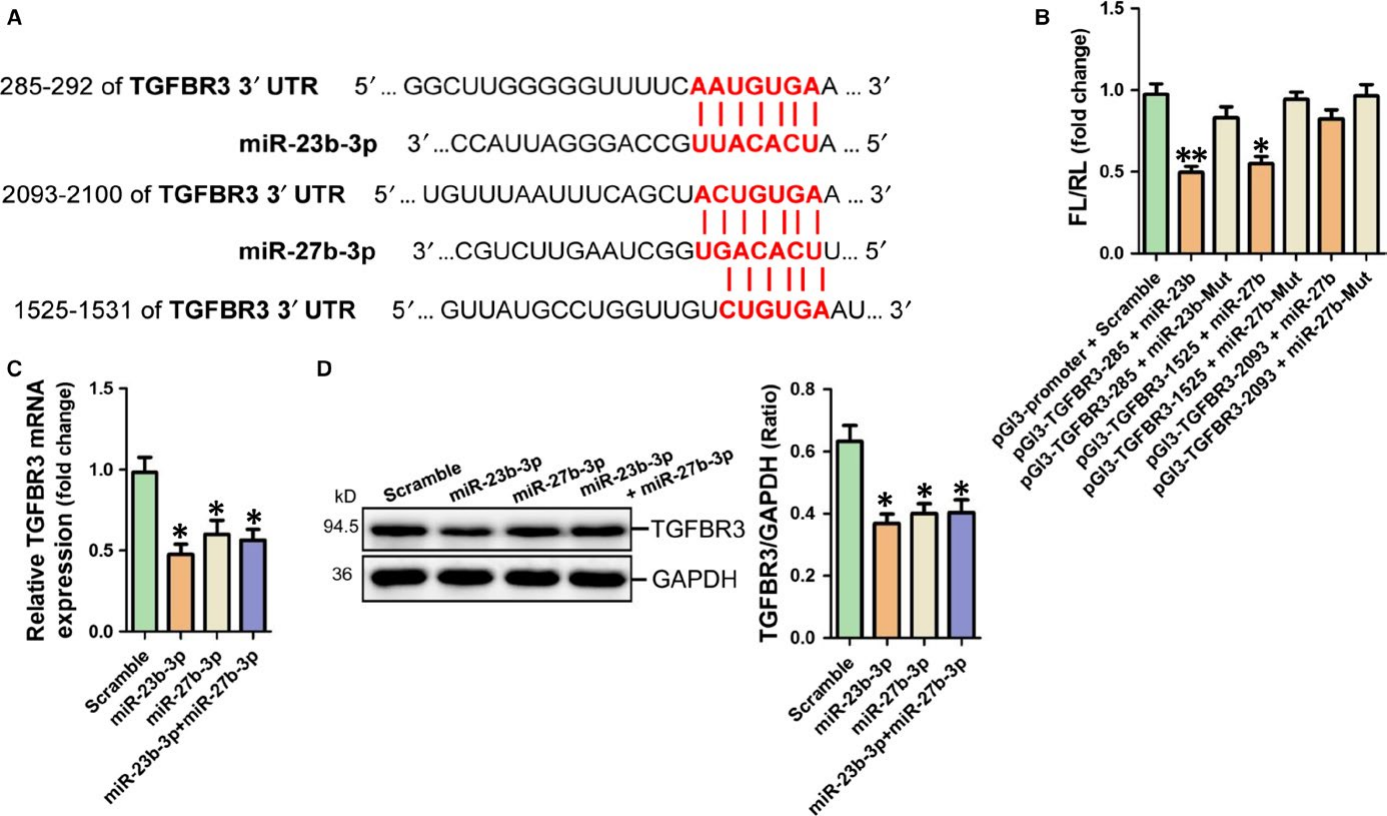
Identification of TGFBR3 as a common target of miR‐23b‐3p and miR‐27b‐3p. A, Predicted miR‐23b‐3p seed and miR‐27b‐3p seed match to the sequence in the 3′UTR of TGFBR3 mRNA. The seed sequences of miR‐23b‐3p and miR‐27b‐3p are UCACAUU and UCACAGU respectively. UGUG in the complementary nucleotide sequences is altered by ACAC to construct the recombinant luciferase reporter plasmids containing the mutant binding sequences of miR‐23b‐3p and miR‐27b‐3p respectively. B, Verification of TGFBR3 as a target gene of miR‐23b‐3p and miR‐27b‐3p by the dual luciferase reporter system. Data are shown as mean ± SEM (n = 3). *P < 0.05, **P < 0.01 vs pGl3‐promoter vector control. The mRNA (C) and protein (D) expression of TGFBR3 in HAFs with transfection of miR‐23b‐3p, miR‐27b‐3p, miR‐23b‐3p combined with miR‐27b‐3p respectively. Data are shown as mean ± SEM (n = 3). *P < 0.05 vs Scramble control

Analysis of the databases Mirdb (www.mirdb.org) and TargetScan‐Vert (www.targetscan.org) showed that TGFBR3 was a potential target gene of miR‐23b‐3p and miR‐27b‐3p respectively. The matching positions for miR‐23b‐3p and miR‐27b‐3p within 3ʹ‐UTR of the targeted TGFBR3 mRNA are shown in Figure 5A. The dual luciferase assay demonstrated that miR‐23b‐3p reduced the luciferase activity by binding to the site of 285‐292 of TGFBR3 3ʹ‐UTR (*P* < 0.01) and miR‐27b‐3p significantly reduced the luciferase activity by binding to the site of 1525‐1531 of TGFBR3 3ʹ‐UTR (*P* < 0.05) (Figure 5B). Moreover, mRNA and protein expression of TGFBR3 was decreased in HAFs with overexpression of miR‐23b‐3p, miR‐27b‐3p and miR‐23b‐3p combined with miR‐27b‐3p respectively (*P* < 0.05 respectively) (Figure 5C,D). However, no significant differences in mRNA and protein expression of TGFBR3 were observed among HAFs with transfection of miR‐23b‐3p, miR‐27b‐3p and miR‐23b‐3p combined with miR‐27b‐3p respectively (Figure 5C,D). TGFBR3 expression at mRNA and protein level was significantly up‐regulated in Ang‐II‐treated HAFs (*P* < 0.01 respectively) (Figure S2A), but not in atrial appendages of AF patients (Figure S2B).

### 3.6 MiR‐23b‐3p, miR‐27b‐3p and TGFBR3 siRNA enhance the expression of fibrosis‐associated gene in HAFs

**Figure 6:**
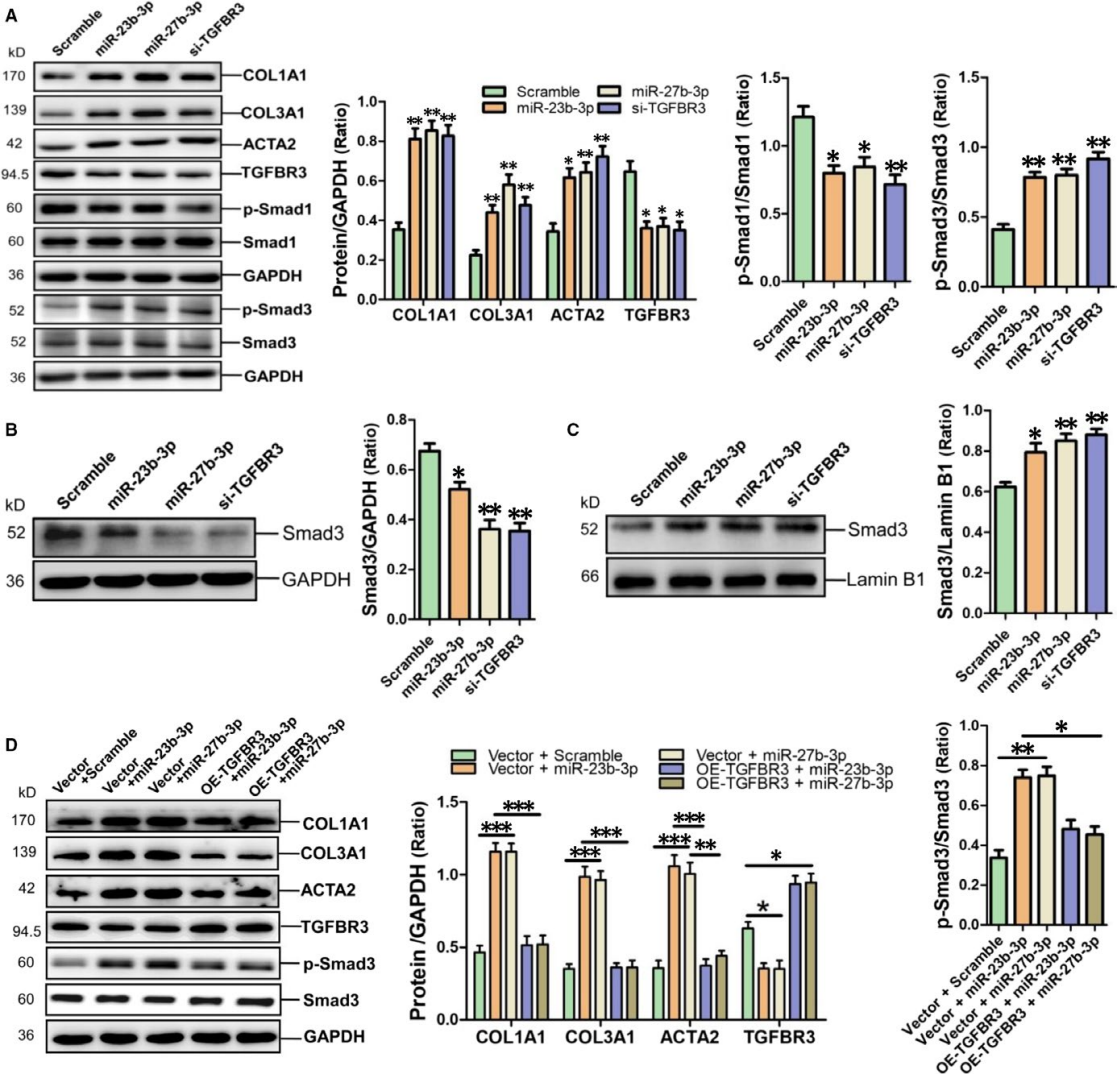
MiR‐23b‐3p and miR‐27b‐3p enhance the expression of COL1A1, COL3A1 and ACTA2 and Smad3 activation in HAFs. A, Protein levels of COL1A1, COL3A1, ACTA2, TGFBR3, p‐Smad1 and p‐Smad3 were detected using western blot assay in HAFs with transfection of miR‐23b‐3p, miR‐27b‐3p and si‐TGFBR3 respectively. Determination of Smad3 in plasma (B) and in the nuclei (C) of HAFs with transfection of miR‐23b‐3p, miR‐27b‐3p and si‐TGFBR3 respectively. Data are shown as mean ± SEM (n = 3). *P < 0.05, **P < 0.01, ***P < 0.001 vs scramble control

MiR‐23b‐3p, miR‐27b‐3p mimic and TGFBR3 siRNA were transfected into HAFs followed by determination of the expression of fibrosis‐related gene. Western blot results showed that miR‐23b‐3p, miR‐27b‐3p and TGFBR3 siRNA efficiently suppressed TGFBR3 expression and increased COL1A1, COL3A1 and ACTA2 expression in HAFs (*P* < 0.05, *P* < 0.01 respectively) (Figure 6A). Consistently, phosphorylated Smad1 (p‐Smad1) was decreased, but p‐Smad3 was increased in HAFs after transfection with miR‐23b‐3p, miR‐27b‐3p and TGFBR3 siRNA respectively (*P* < 0.05, *P* < 0.01 respectively) (Figure 6A). Meanwhile, Smad3 was significantly decreased in the cytoplasm and accumulated in the nuclei of HAFs after transfection with miR‐23b‐3p, miR‐27b‐3p and TGFBR3 siRNA respectively (*P* < 0.05, *P* < 0.01 respectively) (Figure 6B,C). Western blot results showed that adenovirous‐mediated overexpression of TGFBR3 could markedly attenuate the increase of COL1A1, COL3A1 and ACTA2 in HAFs after transfection with miR‐23b‐3p and miR‐27b‐3p respectively (*P* < 0.01, *P* < 0.001 respectively) (Figure 6D). Moreover, TGFBR3 overexpression could significantly alleviate Smad3 activation in HAFs with transfection of miR‐23b‐3p and miR‐27b‐3p respectively (*P* < 0.05 respectively) (Figure 6D).

### 3.7 MiR‐23b‐3p and miR‐27b‐3p are up‐regulated by Ang‐II through the Smad3 pathway

**Figure 7:**
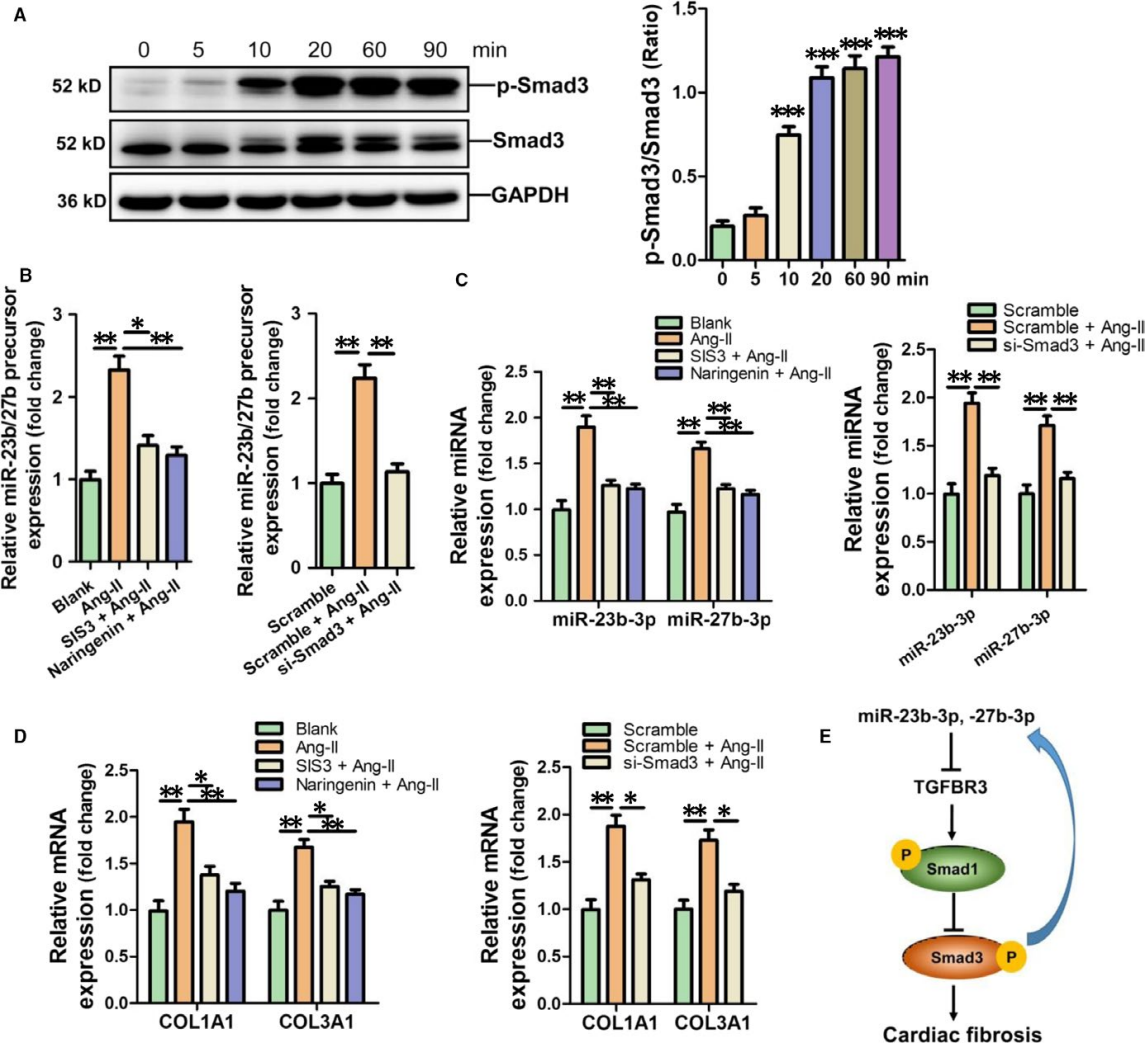
Up‐regulation of miR‐23b‐3p and miR‐27b‐3p in Ang‐II‐treated HAFs through the Smad3 pathway. A, Activation of Smad3 signalling in Ang‐II‐treated HAFs in a time‐course study. Data are shown as mean ± SEM (n = 3). ***P < 0.001 vs 0 min control. The expression of MiR‐23b‐3p and miR‐27b‐3p precursor (B), miR‐23b‐3p and miR‐27b‐3p (C), COL1A1 and COL3A1 mRNA (D) in Ang‐II‐induced HAFs with pre‐treatment with Smad3 inhibitor SIS3 and Naringenin was assessed using RT‐qPCR assay respectively. Data are shown as mean ± SEM (n = 3). *P < 0.05, **P < 0.01. Ang‐II was not used to treat HAFs in the Blank group. E, Schematic diagram of the mechanism whereby Smad3 activation mediates up‐regulation of miR‐23b‐3p and miR‐27b‐3p in HAFs

We first examined the time‐dependent activation of the Smad3 pathway in Ang‐II‐treated HAFs. Our western blot results showed that the phosphorylation level of Smad3 was significantly increased in HAFs at 10 minutes in response to Ang‐II treatment (Figure 7A). Smad3 inhibitors SIS3, Narigenin and Smad3 siRNA were used to suppress Smad3 activation in Ang‐II‐treated HAFs. RT‐qPCR results revealed that treatment with SIS, Narigenin and Smad3 siRNA could prevent Ang‐II‐induced miR‐23b/27b precursor, miR‐23b‐3p and miR‐27b‐3p expression respectively (Figure 7B,C). Moreover, SIS3, Narigenin and Smad3 siRNA could significantly attenuate the increase of COL1A1 and COL3A1 in Ang‐II‐treated HAFs (Figure 7D). Collectively, our results suggest that up‐regulation of miR‐23b‐3p and miR‐27b‐3p in Ang‐II‐treated HAFs results from activation of the Smad3 pathway (Figure 7E).

## 4 DISCUSSION

Increasing evidence shows that interstitial atrial fibrosis is increased in some forms of AF in both humans and animal models.^4^, ^25^, ^26^, ^27^ In this study, we observed the obvious increase of fibrosis in atrial appendage tissue of patients with sinus rhythm, along with the significant elevation of COL1A1, COL3A1 and ACTA2. Meanwhile, the obvious increase of inflammatory factors, such as IL‐1β and CRP, was also found in atrial appendage tissue of AF patients in the current study, which was supported by the previous reports.^28^ Some miRNAs, such as miR‐21^12^, ^13^, ^14^, ^15^ and miR‐26,^16^, ^17^ are involved in the modulation of atrial fibrosis. In this study, we demonstrated that miR‐23b‐3p and miR‐27b‐3p were increased in the human rheumatic atrial appendages and in Ang‐II‐induced HAFs. Functionally, miR‐23b‐3p and miR‐27b‐3p could enhance the expression of fibrosis‐related gene including COL1A1, COL3A1 and ACTA2, in HAFs. These results were supported by previous reports that miR‐23b and miR‐27b enhance myocardial fibrosis.^18^, ^19^, ^20^ Additionally, no significant effects on cell proliferation and migration were observed in HAFs with overexpression of miR‐23b‐3p and miR‐27b‐3p respectively.

The angiotensin system and TGF‐β1 play an important role in atrial fibrosis‐associated with the heart failure models of AF,^29^, ^30^ a mouse model of AF^30^ and in human AF associated with heart failure.^31^ Ang‐II can stimulate NADPH oxidases 2 (Nox2) activity through the TGF‐β1 pathway and inhibition of TGF‐β1 blunts reactive oxygen species (ROS) formation.^32^, ^33^ Therefore, renin–angiotensin–aldosterone system (RAAS)–induced TGF‐β1 pathway and TGF‐β1–induced ROS may contribute to fibrotic remodelling.

As an inhibitory factor of TGF‐β1/Smad3 signalling, TGFBR3 was demonstrated to blunt Tgfbr1/Smad2/3 and potentiate the Acvrl1/Smad1 axis, which was active in lung fibroblasts and antagonized Tgfbr1/Smad2/3 signalling.^34^ A synthetic peptide from TGFBR3, P144, was reported to efficiently inhibit the TGF‐β1‐dependent signalling pathway and collagen type I synthesis in cardiac fibroblasts.^35^ Further studies showed that overexpression of TGFBR3 deactivated the TGF‐β1 pathway by decreasing the expression of TGF‐β1, Smad3 activation, TGFBR1‐TGFBR2 complex formation and inhibiting the pro‐fibrotic microRNA‐21 expression.^24^, ^36^ Therefore, TGFBR3 may act as a protective factor representing a potential therapeutic target for cardiac fibrosis.

Our current study has provided several lines of evidence to support the notion that TGFBR3 is a common target of miR‐23b‐3p and miR‐27b‐3p. First, the in silico prediction indicated that TGFBR3 was a potential target of miR‐23b‐3p and miR‐27b‐3p and the dual luciferase assay revealed that miR‐23b‐3p and miR‐27b‐3p specifically bound to the 285‐292, 1525‐1531 site in the 3′‐UTR of TGFBR3 respectively. We confirmed that miR‐23b‐3p and miR‐27b‐3p mimic inhibited TGFBR3 expression at both mRNA and protein levels in HAFs. In parallel with miR‐23b‐3p and miR‐27b‐3p, TGFBR3 knockdown enhanced the expressions of COL1A1, COL3A1 and ACTA2 in HAFs. Importantly, Smad1 was inactivated, whereas Smad3 was activated and accumulated in the nuclei of HAFs with transfection of miR‐23b‐3p, miR‐27b‐3p and TGFBR3 siRNA respectively. These data indicated that Smad1 inactivation and Smad3 activation mediate the pro‐fibrotic effects of miR‐23b‐3p, miR‐27b‐3p and TGFBR3 siRNA in atrial fibrosis. Meanwhile, the activation of Smad1 through TGFBR3 and the negative modulation between p‐Smad1 and p‐Smad3 were in agreement with the previous report.^37^ Furthermore, TGFBR3 overexpression could significantly block the increase of COL1A1, COL3A1 and ACTA2 in HAFs after transfection with miR‐23b‐3p or miR‐27b‐3p. Therefore, our study demonstrated that the clustered miR‐23b‐3p and miR‐27b‐3p increase Smad3 activation to promote atrial fibrosis by targeting TGFBR3 in HAFs.

The present study revealed that TGFBR3 is a common target of miR‐23b‐3p and miR‐27b‐3p with two different binding sites in 3ʹ‐UTR of TGFBR3 by miR‐23b‐3p and miR‐27b‐3p respectively. However, no synergetic effect on fibrosis‐related gene expression was observed in HAFs with transfection of miR‐23b‐3p combined with miR‐27b‐3p. Moreover, no synergetic effect on mRNA and protein expression of TGFBR3 was found in HAFs with transfection of miR‐23b‐3p combined with miR‐27b‐3p. The potential reason may be associated with that binding of miR‐23b‐3p combined with miR‐27b‐3p in 3ʹ‐UTR of TGFBR3 didn't markedly aggravate TGFBR3 mRNA degradation compare with miR‐23b‐3p only. These results provide evidence that the inhibitory effect of two or more miRNAs on one gene expression was different with that of two or more small interfering RNAs (siRNAs) targeting one gene.

In this study, our data showed that TGFBR3 was significantly increased in Ang‐II‐induced HAFs, but no obvious increase of TGFBR3 was observed in the atrial appendages of AF patients. These results indicated that expression of TGFBR3 in atrial appendages was modulated by more factors, including miRNAs, cytokines and other inflammatory factors.

Smad3 signalling has been shown to participate in cardiac fibrosis.^38^, ^39^ Our present study has confirmed that the Smad3 signalling pathway was activated in Ang‐II‐treated HAFs. We used the Smad3 inhibitors, SIS3 and Naringenin and Smad3 siRNA to verify the participation of Smad3 activation in Ang‐II‐promoted up‐regulation of miR‐23b‐3p and miR‐27b‐3p in HAFs. Therefore, we proposed a positive feedback loop that Smad3 activation modulates miR‐23b‐3p and miR‐27b‐3p expression, which then activate the Smad3 signalling by targeting TGFBR3 in HAFs.

Taken together, our results have demonstrated that miR‐23b‐3p and miR‐27b‐3p were up‐regulated in the atrial appendages of patients with rheumatic AF and in Ang‐II‐treated HAFs. MiR‐23b‐3p and miR‐27b‐3p consistently enhanced fibrosis‐related gene expression with no significant effects on proliferation and migration of HAFs. TGFBR3 was confirmed as a target of miR‐23b‐3p and miR‐27b‐3p and the TGFBR3/Smad1‐Smad3 axis mediated the pro‐fibrotic effect of miR‐23b‐3p and miR‐27b‐3p in HAFs. We also demonstrated that the activation of the Smad3 signalling mediates the up‐regulation of miR‐23b‐3p and miR‐27b‐3p in Ang‐II‐induced HAFs. Therefore, the present study suggests that miR‐23b‐3p and miR‐27b‐3p might be potential targets for AF therapy.

## CONFLICT OF INTEREST

The authors confirm that there is no conflict of interest.
