# Supplementary material for: Novel role of the clustered miR‐23b‐3p and miR‐27b‐3p in enhanced expression of fibrosis‐associated genes by targeting TGFBR3 in atrial fibroblasts
Source: J Cell Mol Med. 2019 Feb 7;23(5):3246–56. doi: 10.1111/jcmm.14211 (PMC6484421; doi:10.1111/jcmm.14211)
Supplement: Supplementary file 1 [file JCMM-23-3246-s001.doc]

Supplementary data

**A B**


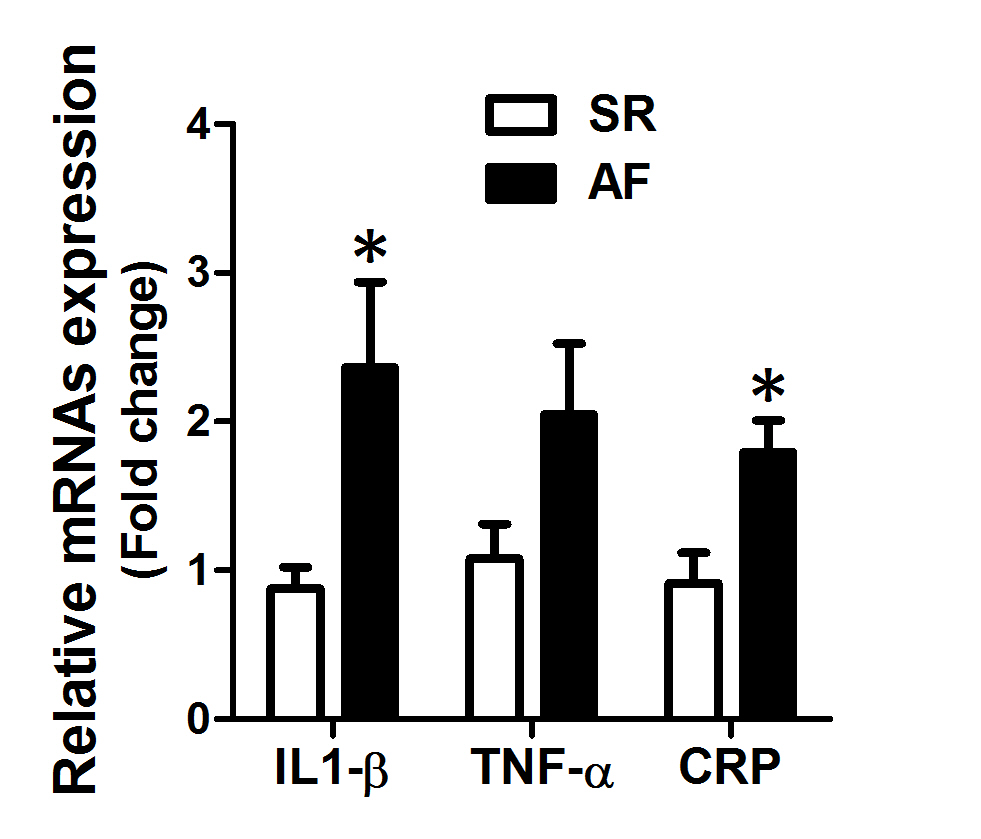

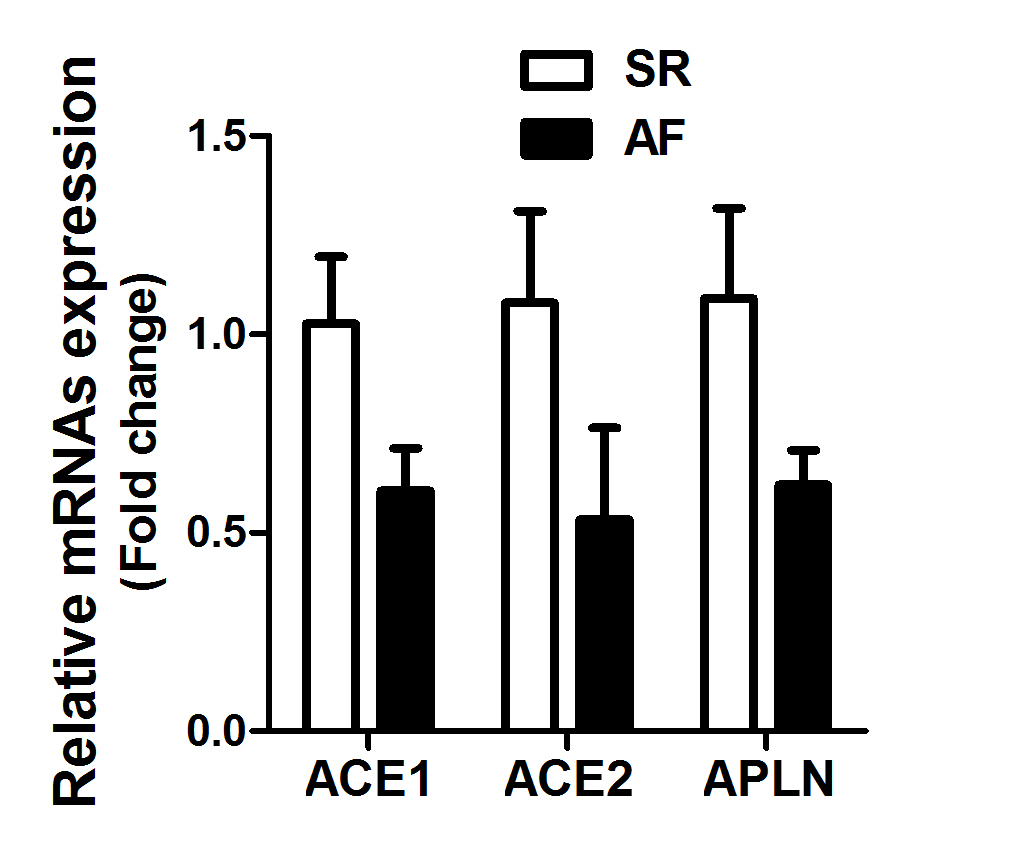


**Figure 1** Detection of concerned genes in atrial appendages of patients with SR or AF. **A**. Expression of inflammation-associated genes by qRT-PCR assay. **B**. Expression of RAAS-associated genes by qRT-PCR assay. Data are shown as mean±SEM; ^*^*p*<0.05 vs. SR control, n=7-9.

**A**

**
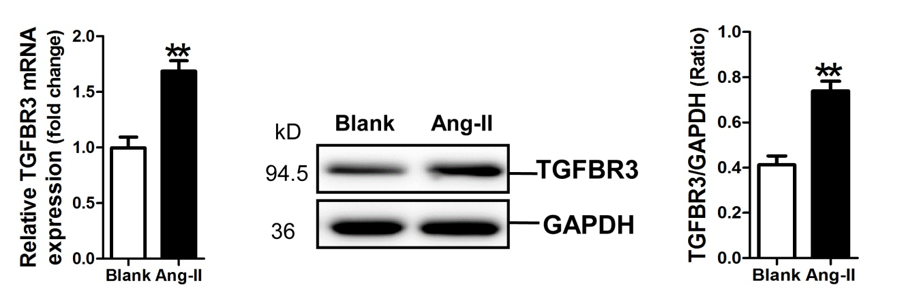
**

**B**


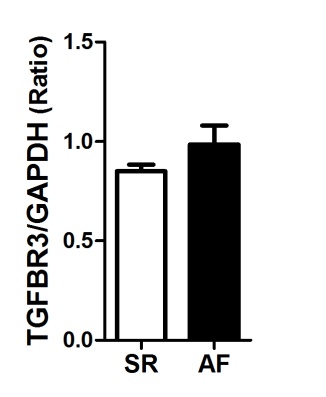

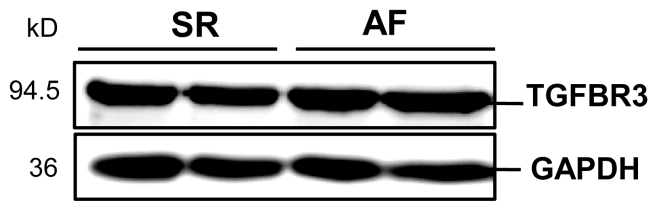

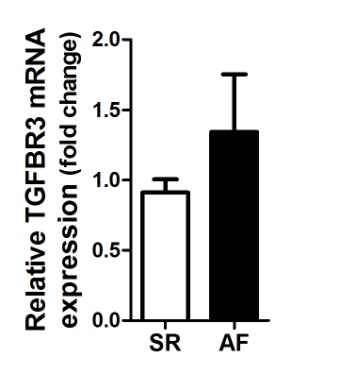


**Figure 2** Detection of TGFBR3 expression in Ang-II-treated HAFs and in atrial appendages of patients with AF. **A**. The mRNA and protein expression of TGFBR in Ang-II-treated HAFs. Data are shown as mean±SEM; ^**^*p*<0.01 vs. Blank control, n=3. **B**. The mRNA and protein expression of TGFBR in atrial appendages of patients with SR or AF. Data are shown as mean±SEM, n=7-9.


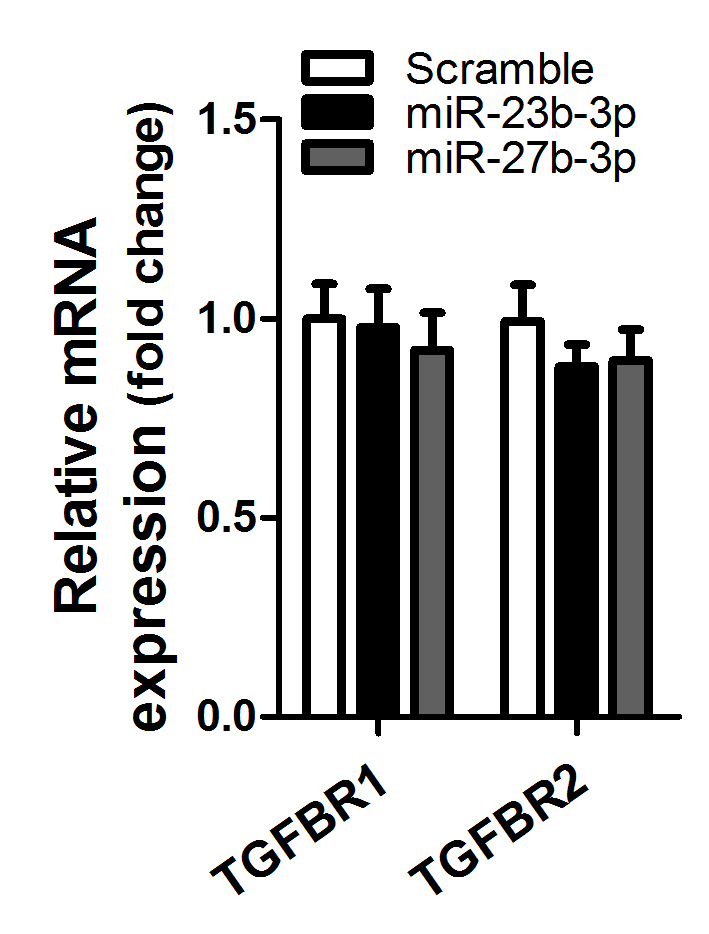


**Figure 3** Detection of TGFBR1 and TGFBR2 mRNA level in HAFs with transfection of scramble, miR-23b-3p and -27b-3p, respectively. Data are shown as mean±SEM, n=3.
